# Combined treatment with glucosamine and chondroitin sulfate improves rheumatoid arthritis in rats by regulating the gut microbiota

**DOI:** 10.1186/s12986-023-00735-2

**Published:** 2023-04-04

**Authors:** Xuesong Wang, Dongsong Liu, Dan Li, Jiai Yan, Ju Yang, Xiaohui Zhong, Qin Xu, Yuanze Xu, Yanping Xia, Qinyue Wang, Hong Cao, Feng Zhang

**Affiliations:** 1 Affiliated Hospital of Jiangnan University, Wuxi, China; 2 School of Medicine, Nantong University, Nantong, China; 3 Wuxi School of Medicine, Jiangnan University, Wuxi, China

**Keywords:** Glucosamine, Chondroitin sulfate, Gut microbiota, Rheumatoid arthritis

## Abstract

**Background:**

To investigate the ameliorative effects of glucosamine (GS), chondroitin sulphate (CS) and glucosamine plus chondroitin sulphate (GC) on rheumatoid arthritis (RA) in rats, and to explore the mechanism of GS, CS and GC in improving RA based on the gut microbiota.

**Methods:**

RA rat models were effectively developed 14 days after CFA injection, and then garaged with GS, CS and GC. Body weight and paw volume of rats were monitored at multiple time points at the beginning of CFA injection. Until D_36_, serum and ankle tissue specimens were used to measure levels of circulating inflammatory factors (TNF-α, IL-1β, MMP-3, NO and PGE_2_) and local inflammatory indicators (TLR-4 and NF-κB). On D_18_, D_25_, and D_36_, intergroup gut microbiota was compared using 16S rRNA gene sequencing and bioinformatics analysis. We also performed the correlation analysis of gut bacteria, joint swelling and inflammatory indicators.

**Results:**

GC, rather than GS and CS, could reduce right paw volumes, levels of TLR-4 and NF-κB in synovial tissues. In addition, enriched genera in RA model rats screened out by LEfSe analysis could be inhibited by GC intervention, including potential LPS-producing bacteria (*Enterobacter, Bacteroides*, *Erysipelotrichaceae_unclassified* and *Erysipelotrichaceae_uncultured*) and some other opportunistic pathogens (*Esherichia_Shigella, Nosocomiicoccus, NK4A214_group, Odoribacter, Corynebacterium* and *Candidatus_Saccharimonas*.etc.) that positively correlated with pro-inflammatory cytokines, right paw volume, and pathology scores. Furthermore, the gut microbiota dysbiosis was observed to recover before alleviating joint swelling after interventions.

**Conclusions:**

GC could inhibit potential LPS-producing bacteria and the activation of TLR-4/NF-κB pathway in RA rats, thus alleviating RA-induced joint injury.

**Supplementary Information:**

The online version contains supplementary material available at 10.1186/s12986-023-00735-2.

## Background

Rheumatoid arthritis (RA) is a chronic inflammatory disease characterized by synovial inflammation and is most clinically significant in terms of damage to the joints, manifested by progressive damage to the articular cartilage, causing swelling, pain, and stiffness, and eventually leading to impaired joint mobility [1, 2]. With 0.5%-1% of the world's population are suffering from RA, which is a considerable burden on individuals and society [3]. The specific pathological mechanisms involved in RA remain unclear but delayed joint dysfunction has become the mainstay of treatment for RA [4]. Most of the clinical drugs currently applied in the treatment of RA come with more or fewer side effects. As a result, attention has been focused on finding functional nutritional foods that can complement or even replace the effects of drugs.

Glucosamine (GS) and chondroitin sulfate (CS) are synthesized from natural substances and are widely marketed as functional nutritional foods for joint protection. GS is a naturally occurring monosaccharide that is a key component of glycosaminoglycans in the extracellular matrix of articular cartilage and other connective tissues. CS, on the other hand, is a complex polysaccharide consisting of repeating disaccharide chains (glucuronide and n-acetylgalactosamine), which are also structural components of articular cartilage, providing it with resistance to compression. In recent years, glucosamine plus chondroitin sulfate (GC) have been increasingly used in the improvement of osteoarthritis [5–7].. However, no studies had shown whether GS, CS and GC can improve RA. Intriguingly, GS and CS modulate the abundance of *Prevotella*, Bacteroidaceae and Clostridiaceae, which have been shown to be enriched in RA [8–11]. This suggests that the gut microbiota may play a role in the effect of GS, CS and GC on RA.

Adjuvant arthritis (AA) was used to confirm the efficacy of GS, CS and GC on RA, respectively. In addition, 16S rRNA gene sequencing and bioinformatics analysis were used to confirm the existence of a pathway by which GS, CS and GC could also regulate the gut microbial composition and thereby ameliorating the joint injury in RA. In this study, the gut microbiota was used to interrelate functional nutritional foods and diseases such as RA, which is also expected to provide a more novel perspective for future related studies.

## Methods

### Animals

Adult male SD rats weighing approximately 270 g were maintained in a standard laboratory environment. Two standard cages were loaded with a total of seven rats in groups of five, all of which were provided with the same potable water and standard rat chow for ad libitum feeding. Each SD rat was allowed a fourteen-day window to acclimatize to the laboratory environment before the experiment was conductedfig.

### Reagents

Complete Freund’s adjuvant (CFA) containing 10 mg/ml heat-killed Mycobacterium tuberculosis H37 RA (non-viable) was purchased from Chondrex Inc. (USA) for induction of AA model. GS and CS were purchased from Jiaxing Hengjie Biopharmaceutical Co., LTD. The corresponding ELISA kits were obtained from Hushang Biotechnology Co. (Shanghai, China). Antibodies against nuclear factor kappa B (NF-κB), toll-like receptor 4 (TLR-4), and HRP-conjugated secondary anti-rat and anti-rabbit antibodies were purchased from Proteintech Group, Inc., USA.

### Induction of AA model

AA, previously been used as a classic model for RA, can easily be induced in susceptible strains of rats by injection of 0.1 ml suspension of heat-killed Mycobacterium tuberculosis incomplete Freund’s adjuvant at a concentration of 10 mg/ml, intradermally at the base of the tail [12].

### Study design

**Fig. 1:**
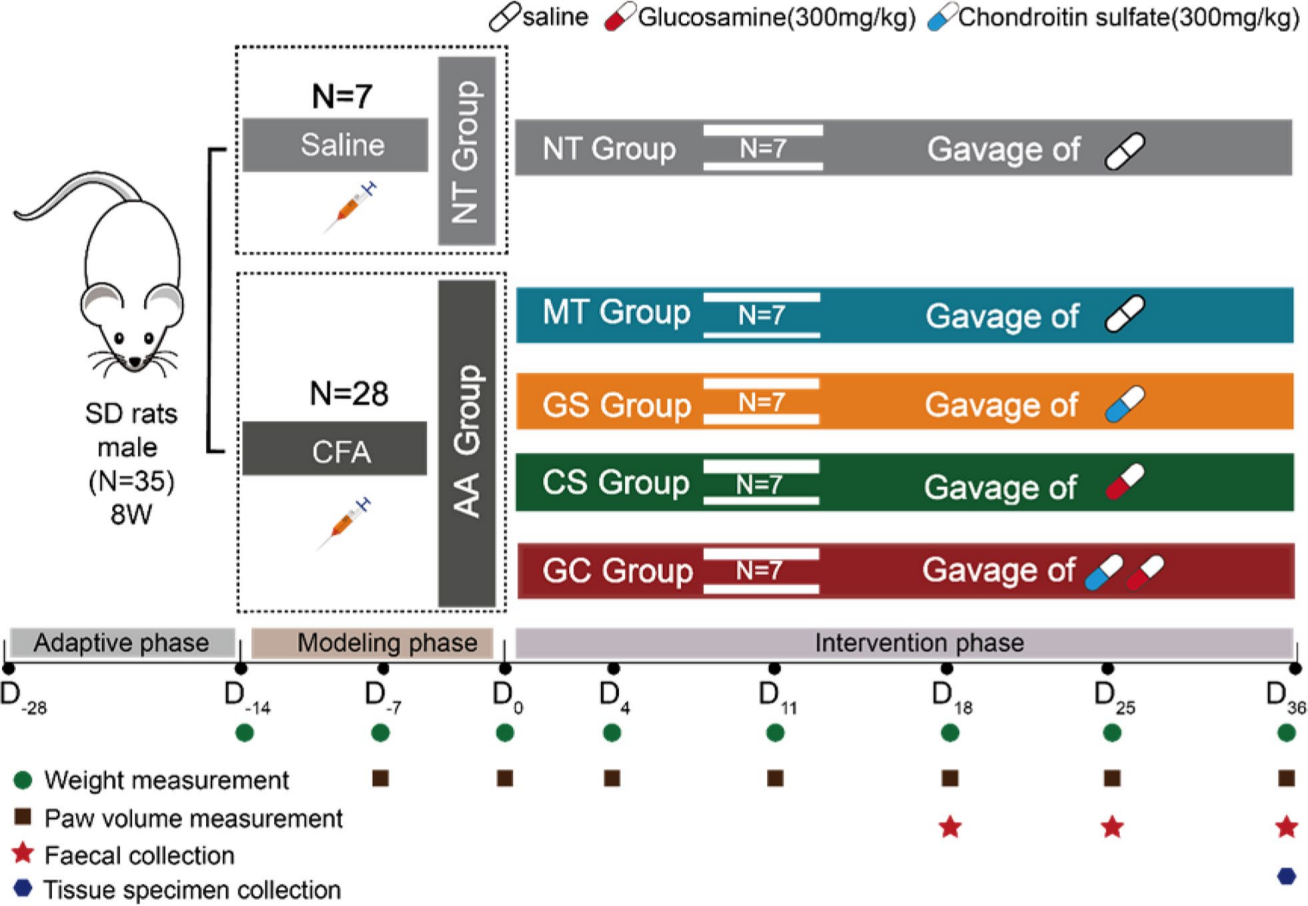
Flow chart of the study. NT group, normal test group. AA group, CFA-injected group. MT group, model group. GS group, glucosamine group. CS group, chondroitin sulfate group. GC group, glucosamine combined with chondroitin sulfate group. D_0_, the day of an intervention or the model successful day

All rats were assigned into 5 groups of 7 rats each as follows (Fig. 1): Normal test (NT) group, non-arthritic healthy control rats receiving a daily oral dose of the saline. Model test (MT) group, AA rats receiving a daily oral dose of the saline. GS group, AA rats treated daily oral dose of GS (300 mg/kg) [13]. CS group, AA rats treated daily oral dose of CS (300 mg/kg) [14]. GC group, AA rats treated daily oral dose of GS (300 mg/kg) plus CS (300 mg/kg). In this study, the above intervention was performed on AA model successful day (D_0_) and remained in place for day 36 of the intervention until the end.

### Assessment of arthritis

Basal right hind paw volume of the rats using a digital plethysmometer (IITC Inc. Life Science, Woodland Hills, CA, USA). The progression of arthritis was evaluated by measuring the right hind paw volume on D_0_, D_4_, D_11_, D_18_, D_25_, and D_36_ (the day of an intervention or the model successful day is recorded as “D_0_”).

### Serum cytokines measurement

At D_36_, all blood specimens were collected through the posterior orbital plexus vein and placed in sterile EP tubes. Serum was obtained by centrifugation at 3500 rpm for 10 min after being placed in a refrigerator overnight at 4 °C and stored in units of 200 µl at − 80 °C for further analysis. Tumor necrosis factor (TNF)-α, Interleukin (IL)-1β, IL-10, prostaglandin E_2_ (PGE_2_), matrix metalloproteinase (MMP)-3 and nitric oxide (NO) were determined through enzyme immunosorbent assay (ELISA) or electrochemical methods according to the relevant procedures.

### Histopathological parameters

At D_36_, right ankle joints were removed and immediately fixed with 4% paraformaldehyde for histological examination. Then, the right ankle joints were decalcified with 10% calcium nitrate, dehydrated with a gradient, series of ethanol, washed twice with xylene and embedded in paraffin. Finally, 4 μm slices were sectioned and stained with hematoxylin-eosin (H&E) for histopathological analysis and examined using light microscopy. The severity of arthritis in the joint was scored from 0 to 3 according to inflammatory cell infiltration, Pannus formation, synovial hyperplasia, destruction of articular cartilage and bone, and the total score of each mouse was calculated [15].

### Immunohistochemistry

4 μm paraffin sections were baked at 60 °C for 30 min, then deparaffinized and rehydrated in xylene and descending gradient alcohol solution, followed by PBS rinsing. Sections were placed in citric acid solution (0.1 mol/L and pH 6.0) repair solution for antigen repair. Endogenous peroxidase activity was blocked by 3% H_2_O_2_ and antigen closure was performed using 5% BSA as the closure solution. After incubation in blocking buffer, these sections were treated with primary antibody against TLR-4 and NF-κB at a dilution of 1:300, respectively. Sections with sufficient peroxidase-labeled polymer secondary antibodies were re-incubated at room temperature. Finally, DAB was used as chromogen and these sections were counterstained with hematoxylin.

### 16S rRNA gene sequencing and bioinformatics analysis

Microbial community genomic DNA was extracted from all fecal samples using the E.Z.N.A.® soil DNA Kit (Omega Bio-Tek, Norcross, GA, U.S.) according to the manufacturer’s instructions. The DNA extract was checked on 1% agarose gel, and DNA concentration and purity were determined with NanoDrop 2000 UV-vis spectrophotometer (Thermo Scientific, Wilmington, USA). The region V3-V4 of the bacterial 16S rRNA gene was amplified with primer pairs 338F: ACTCCTACGGGAGGCAGCAG’ and 806R: GGACTACHVGGGTWTCTAAT by an ABI GeneAmp® 9700 PCR thermocycler (ABI, CA, USA). Purified amplicons were pooled in equimolar and paired-end sequenced on an Illumina MiSeq PE300 platform (Illumina, San Diego, USA) according to the standard protocols by HonSunBio Technology Co. Ltd. (Shanghai, China).

Paired-end reads were quality-filtered by fastp version 0.20.0 and merged by FLASH version 1.2.11 [16, 17]. Operational taxonomic units (OTUs) with 97% similarity cutoff were clustered using UPARSE version 7.1, and chimeric sequences were identified and removed [18]. The taxonomy of each NAÏVE representative sequence was analyzed by RDP Classifier version 2.2 against the 16S rRNA gene database (e.g. Silva v138) using a confidence threshold of 0.7 [19]. The overall structure of the microbial community was analyzed by weighted UniFrac Principal Coordinate analysis (PCoA). The linear discriminant analysis effect size (LEfSe, Galaxy version 1.0) was performed to identify the significant differences between groups [20, 21].

### Statistical analysis

Data were expressed as mean ± standard error of the mean (SEM). The significance of differences between the groups was determined using one-way ANOVA or T-test using GraphPad Prism version 8.0.2 (263). *P* values < 0.05 were considered significant.

## Results

### Changes in weight of rats in each group

Rats in CFA-injected group were prone to lose weight when compared to rats in the NT group, although no significant difference was observed (Additional file 1: Fig. 1). During D_0_ to D_36_, no significant differences in body weight were observed among five groups.

### Joint swelling was relieved by interventions

**Fig. 2:**
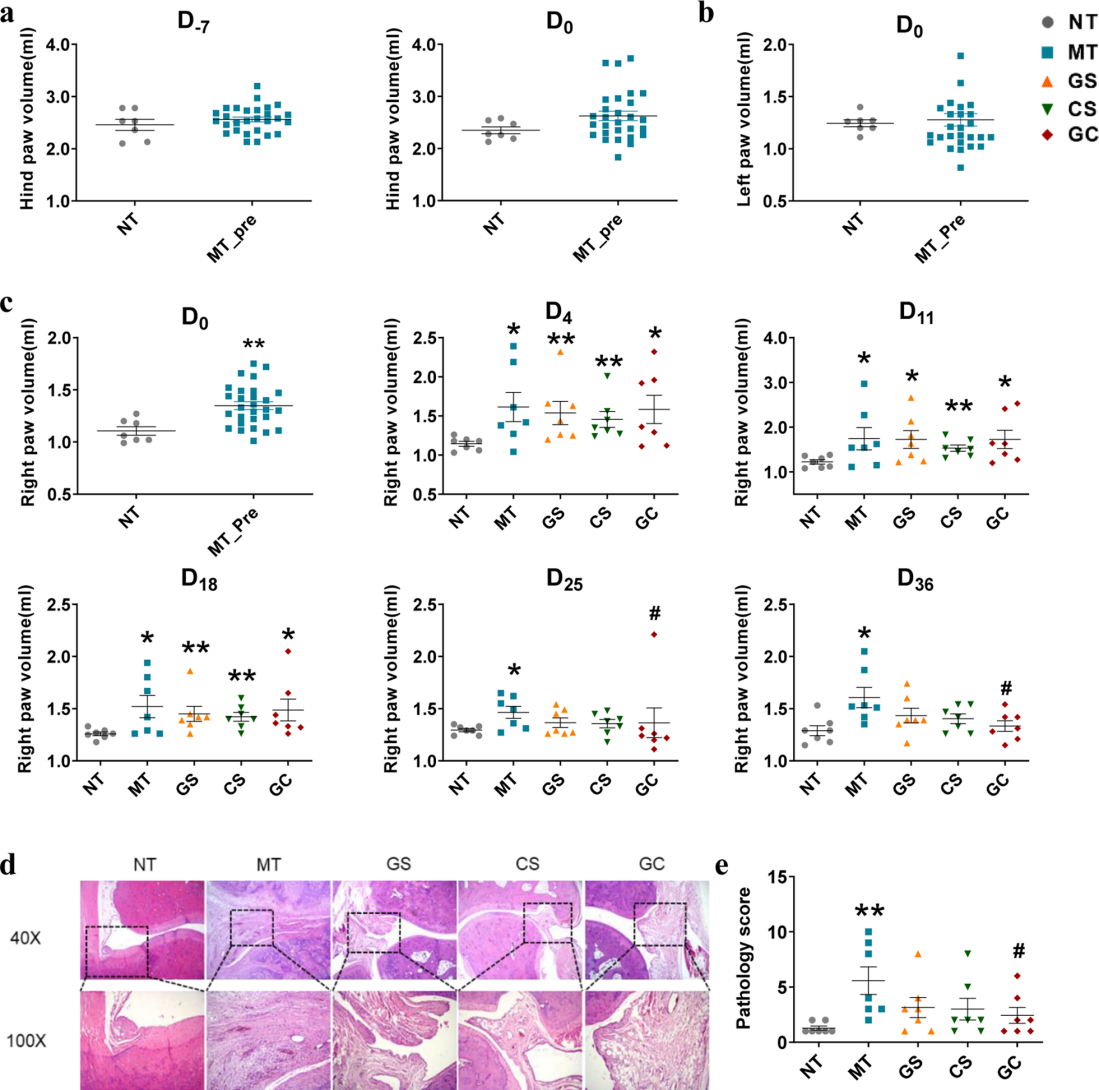
Changes of joint swelling in rats. **a** Hind paw volume of rats in each group at D_-7_ and D_0_. **b** Left paw volume of rats in each group at D_0_. **c** Swelling degree of right hind paw volume of rats in each group at several time points. **d** Histologic images of the talus bone and cartilage surface of ankle joints stained with H&E (40X & 100X magnification) at D_36_. **e** Pathological scores were calculated. (MT_Pre, the group of pre-intervention modeling. *p < 0.05 and **p < 0.01 compared with NT group. #p < 0.05 compared with MT group)

Joint swelling is one of the most important features of the AA model. At D_-7_, we found no significant difference in paw volume between the NT group and the MT group (Fig. 2a, *P*= 0.423). Although there was no significant difference in hindfoot and left paw volumes in the MT group compared to the NT group until D_0_, a significant difference appeared in the right paw volume (Fig. 2b, c, *P* = 0.002), indicating that the AA model was effectively established. The tumefaction degrees of paws swelling were diminished in the groups treated with GS, CS and GC on the end of our study (Additional file 2: Fig. 2). From D_0_ to D_18_, we found no significant difference in the right paw volume of rats between the MT group and three intervention groups, but the right paw volume of rats in the CFA-injected group was significantly high than that of rats in the NT group (Fig. 2c). At D_25_, there was significant difference in right paw volume between rats in the GC group compared with the MT group (Fig. 2c), indicating that GC intervention significantly improved joint swelling. Right paw volumes in the GS and CS groups were not significantly different with the MT and NT group, while fall in between the NT group and the MT group. This finding implied that GS and CS also alleviate joint swelling to some extent, although no statistically significant difference was found.

### Joint pathology scores decreased by interventions

The HE-stained pathological sections of the right hind ankle joint of each group of rats are shown in Fig. 2d. Ankle joints of rats in the MT group exhibited pathological features such as inflammatory cell infiltration, severe synovial hyperplasia and the destruction of articular cartilage. Pathological scores in the GS and CS groups were not significantly different with the MT and NT group, while fall in between the NT group and the MT group. This finding indicated that GS and CS groups could tend to improve the histological damage of RA model rats. Rats in the GC group had considerably lower pathology scores than the MT group (Fig. 2e, *P*<  0.01), which indicated that GC group showed a significant improvement of pathological scores.

### Changes in circulating inflammatory cytokines after interventions

**Fig. 3:**
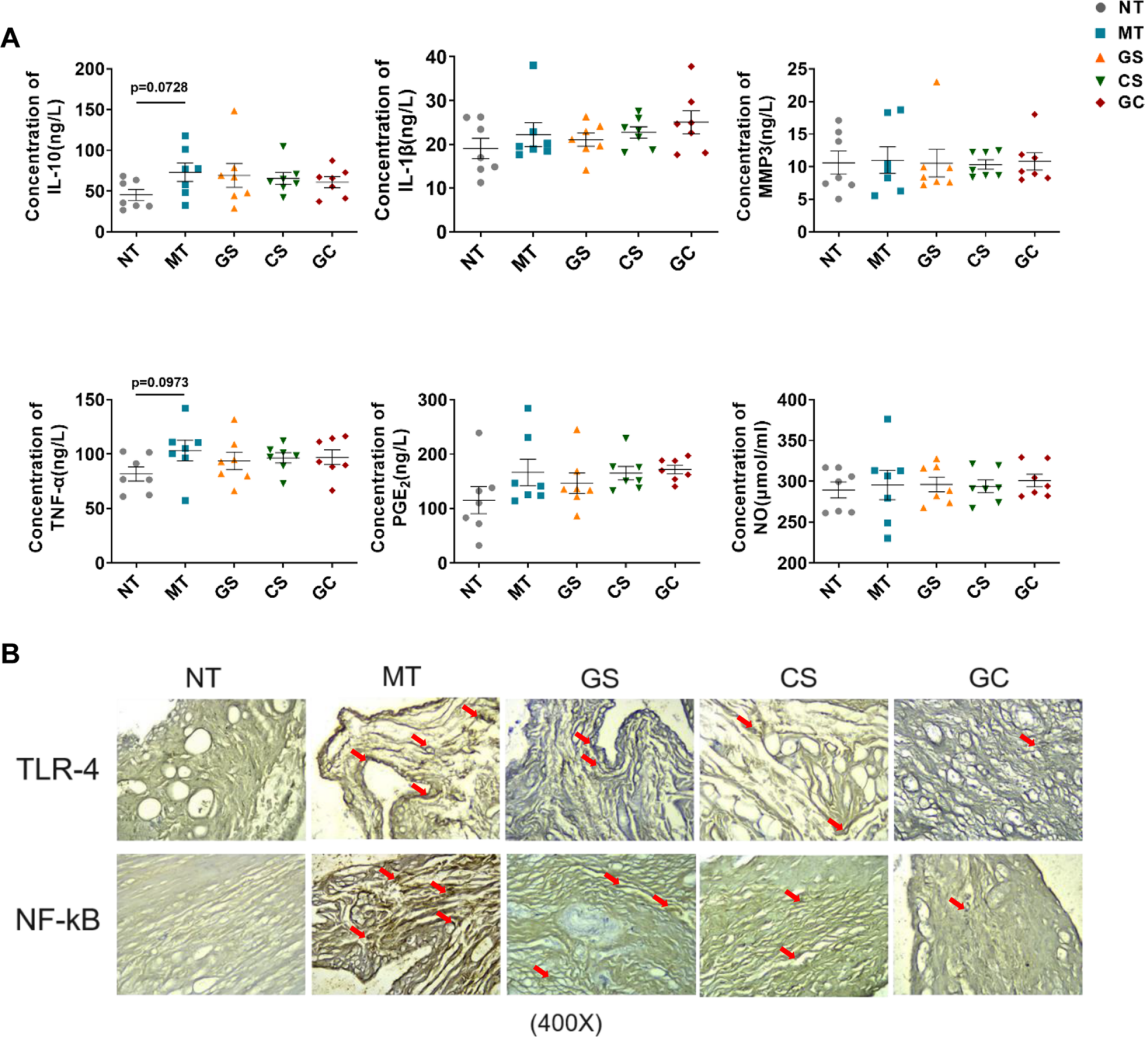
Changes of inflammatory indicators. **a** Circulating inflammatory cytokines were determined through ELISA or electrochemical methods at D_36_ according to the relevant procedures. (*p < 0.05 and **p < 0.01 compared with NT group; #p < 0.05 compared with MT group) **b** Local inflammatory indicators** (**the expression of TLR-4 and NF-κB) in synovial tissues was detected by immunohistochemical methods at D_36_. The red arrows represent dye enrichment points

Serum TNF-α, IL-1β, MMP-3, NO and PGE_2_ are pro-inflammatory factors, while IL-10 is an anti-inflammatory factor. At D_36_, a tendency for the level of serum TNF-α of rats in the MT group to be higher than in the serum of rats in the NT group (Fig. 3a, *P*=  0.0973), while no significant differences in other inflammatory indexes were observed. In addition, the expression level of serum IL-10 tended to be higher in the MT group than in the NT group (Fig. 3a, *P*= 0.0728), and no statistically significant difference was observed among three intervention groups and MT group.

### Expression levels of TLR-4 and NF-κB in synovial tissues decreased after interventions

Immunohistochemical methods were used to detect expression levels of TLR-4 and NF-κB in the synovial tissues in rats. (Fig. 3b) TLR-4 and NF-κB were highly expressed in the synovia tissues of rats in the MT group, and oral administration of GS or CS could tend to reduce the expression levels of TLR-4 and NF-κB. Notably, oral GC could significantly inhibit the expression of TLR-4 and NF-κB in the synovial tissues in RA rats.

### Changes in the composition of the gut microbiota after interventions

**Fig. 4:**
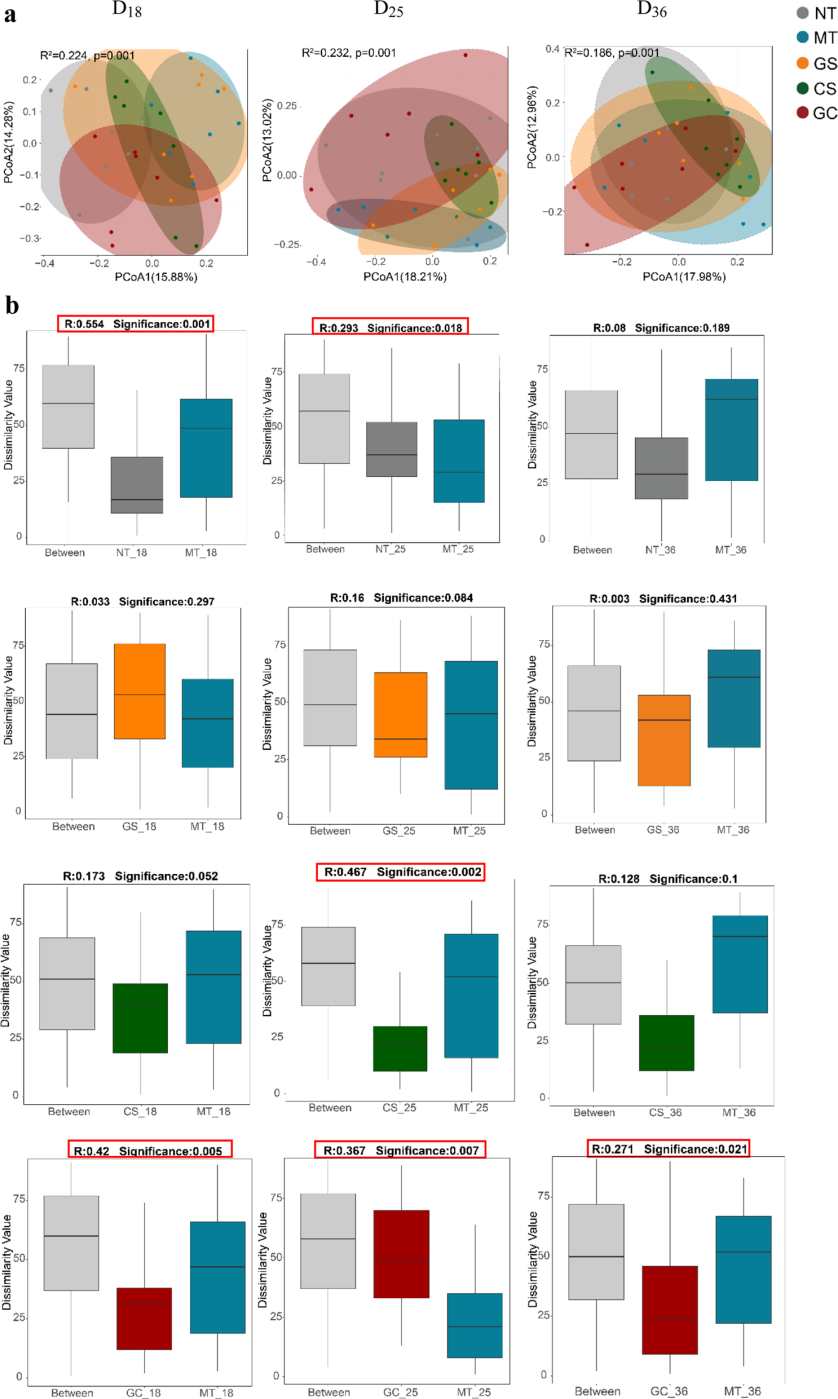
Differences in the gut micrbiota composition of rats in each group. **a** PCoA plots show the horizontal coordinate (PCoA1) and the vertical coordinate2 (PCoA2) representing the suspected influences on the bias of microbial composition between groups, respectively. Each point represents a sample. Different colors represent different groups. **b** The difference in the gut microbiota between MT group and NT, GS, CS and GC group respectively at three time points. *P* < 0.05 was considered to be statistically significantly different

16S rRNA gene sequencing was used to detect fecal microbiota at D_18_, D_25_, and D_36_ in rats in each group. Results of PCoA showed significant differences among five groups at all three time points (Fig. 4a , R^2^= 0.224, *P* = 0.001; R^2^ = 0.232, *P* = 0.001; R^2^ = 0.186, *P* = 0.001). In addition, a significant difference in the microbiota structure occurred between the MT and NT group at D_18_ and D_25_ (Fig. 4b, *P* < 0.05), indicating the gut microbiota dysbiosis occurred in CFA-injected rats. At D_36_, no significant difference in microbiota structure was observed between the MT and NT groups (Fig. 4b, *P* = 0.189), implying that the structure of the gut microbiota in the MT group gradually approached that of the NT group after a relatively long time. In addition, it showed that no significant differences were observed in the microbiota structure in the GS and MT group from three time points. A significant difference was found only in the CS group compared to the MT group at D_25_ (Fig. 4b, *P*  = 0.002), and no significant differences were discovered at D_18_ and D_36_. Notably, there was a significant difference in the gut microbiota structure between the GC and MT group at D_18_, D_25_ and D_36_ (Fig. 4b, *P* < 0.05), suggesting that oral supplementation with GC has a durable ability to alleviate gut microbiota disorders accompanied by the pathological process of RA.

### Changes in the key gut bacteria after interventions

**Fig. 5:**
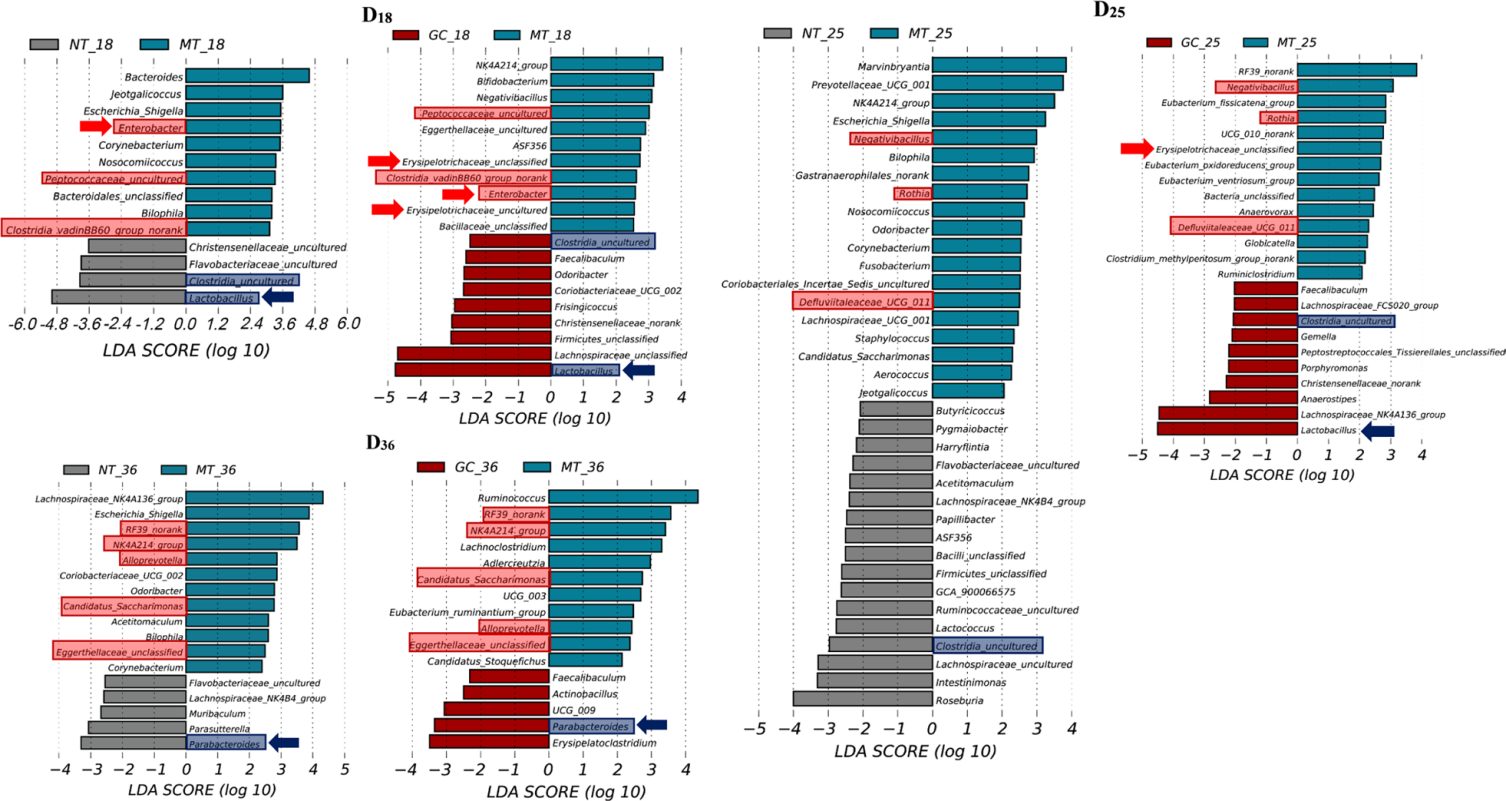
Plots of LEfSe analysis of differential key genera between NT and MT groups and between MT and GC groups at three time points. Red boxes, key genera enriched in the MT group and reversed after GC intervention. Blue boxes, key genera enriched in both NT and GC groups. Red arrows, key genera positively correlated with LPS. Blue arrows, key genera negatively correlated with LPS

To further reveal changes in the key gut bacteria in rat group, LEfSe analysis was used to identify bacterial taxa that differed in abundance between groups, considering only those with an LDA score of > 2. As only GC showed a statistically significant improvement in symptoms compared to the MT group, we focused on the key differential genera that existed between the MT group and the NT and GC groups, respectively, at D_18_, D_25_ and D_36_ (Fig. 5).

On the one hand, key genus number in NT group was 4, 17 and 5, while in MT group was 10, 19 and 12 at D_18_, D_25_ and D_36_, respectively. Among them, *Escherichia_Shigella* and *Bilophila* continued to show high abundance in the MT group at D_18_, D_25_ and D_36_.

On the other hand, at D_18_, the abundance of *Enterobacter*, *Peptococcaceae_uncultured* and *Clostridia_vadinBB60_group_norank* increased and the abundance of *Lactobacillus* and *Clostridia_ uncultured* decreased in the MT group compared to the NT group. Meanwhile, GC intervention reversed the abundance of the above genera. Moreover, *Negativibacillus*, *Rothia* and *Deffuviitaleace_UCG_011* showed significantly high abundance, while *Clostridia_uncultured* showed low abundance in the MT group at D_25_. GC intervention for 25 consecutive days reversed the changes in the abundance of these genera. Similarly, GC intervention for 36 days reduced the abundance of five increased genera including *RF39_norank*, *NK4A214_group*, *Alloprevotella*, *Candidatus_Saccharimonas* and *Eggerthellaceae_unclassified* in the MT group, while increasing the abundance of *Parabacteroides* inhibited by CFA injection. Notably, at D_18_ and D_25_, GC intervention significantly increased the abundance of *Lactobacillus* which was considered to be negatively associated with lipopolysaccharide (LPS), and decreased the abundance of LPS-producing genera (e.g., *Enterobacter*, *Erysipelotrichaceae_g_uncultured* and *Erysipelotrichaceae_g_unclassified*) [22, 23].

### Correlation analysis of gut microbiota, joint swelling and inflammatory indicators

**Fig. 6:**
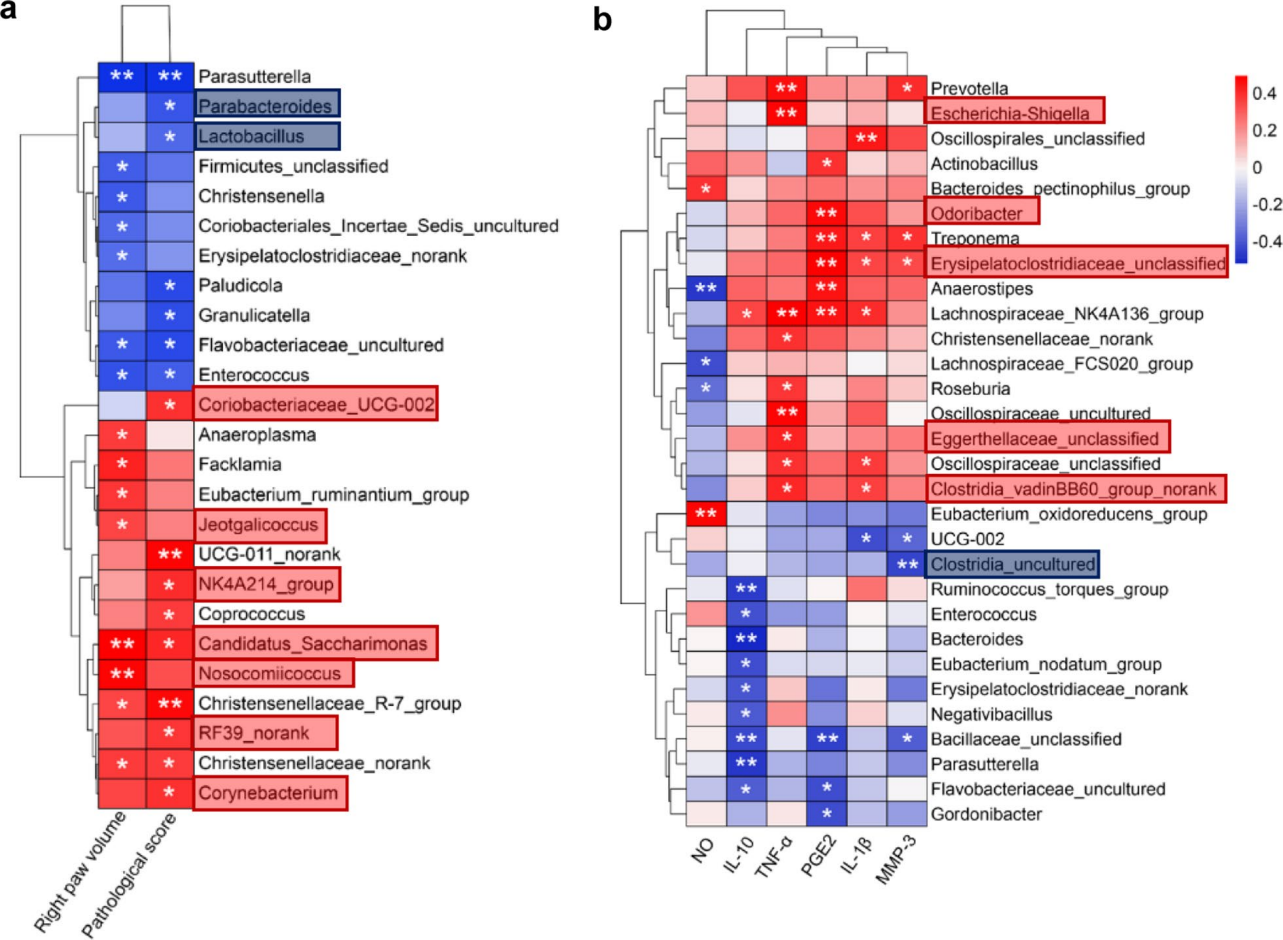
Heatmap of correlations between the gut microbiota, clinical indicators **a** and serological indicators **b** in different groups at the end of study. The correlation coefficients were calculated using Spearman correlation test. Boxes are labeled with the key genera selected in the LEfSe analysis. *p < 0.05, **p < 0.01. Red color, positive correlation. Blue color, negative correlation. Red boxes, key genera enriched in the MT group and reversed after GC intervention. Blue boxes, key genera enriched in both NT and GC groups

We analyzed the correlation between joint swelling indicators and gut bacteria in rats (Fig. 6). We found that right paw volume was negatively correlated with the abundance of *Firmicutes_unclassified* (r = − 0.378, *P* < 0.05), but positively correlated with the abundance of *Jeotgalicoccus* (r = 0.335, *P* < 0.05), *Nosocomiicoccus* (r = 0.452, *P* < 0.01) and *Candidatus_Saccharimonas* (r = 0.470, *P* < 0.01). In addition, pathology scores were negatively correlated with the abundance of *Lactobacillus* (r = − 0.350, *P* < 0.05) and *Parabacteroides* (r = − 0.391, *P* < 0.05), but positively correlated with the abundance of *NK4A214_group* (r = 0.380, *P* < 0.05), *Corynebacterium* (r = 0.372, *P* < 0.05), *RF39_norank* (r = 0.360, *P *< 0.05)*, Candidatus_Saccharimona* (r = 0.390, *P* < 0.05) and *Coriobacteriaceae_UCG_002* (r = 0.365, *P* < 0.05). In addition, we also discovered that serum TNF-α was positively correlated with the abundance of *Esherichia_Shigella* (r = 0.479, *P* < 0.01), *Eggerthellaceae_unclassified* (r = 0.417, *P* < 0.05) and *Clostridia_vadinBB60_group_norank* (r = 0.406, *P* < 0.05). Serum IL-1β was positively correlated with the abundance of *Erysipelatoclostridiaceae_unclassified* (r = 0.343, *P* < 0.05) and *Clostridia_vadinBB60_group_norank* (r = 0.352, *P* < 0.05). Serum PGE_2_ was positively correlated with the abundance of *Odoribacter* (r = 0.461, *P* < 0.01) and *Erysipelatoclostridiaceae_unclassified* (r = 0.492, *P* < 0.01). Serum MMP-3 was negatively correlated with the abundance of *Clostridia_uncultured* (r = − 0.441, *P* < 0.01), but positively correlated with the abundance of *Erysipelatoclostridiaceae_unclassified* (r = 0.339, *P* < 0.05). Serum NO was negatively correlated with the abundance of *Lachnospiraceae_FCS020_group* (r = − 0.425, *P* < 0.05).

## Discussions

RA was an autoimmune disease that affects the joints primarily, causing cartilage and bone degeneration as well as functional disability [1, 2]. TLRs and many endogenous TLR ligands were present in inflamed joints. Several microbial TLR ligands had been identified to display a synovial fibroblast response in RA, with TLR-4 being more responsive to TLR ligation in RA than other TLRs. [12] TLRs can be activated by LPS to increase the expression of M1-type macrophages in the synovial membrane [24, 25]. M1-type macrophages could release several destructive factors such as IL-1β, IL-6, and TNF-α for subsequent cartilage [25]. In addition, TNF-α, IL-1β, IL-6, and MMPs were released by activated immune cells (macrophages, mast cells, and neutrophils, etc.), which encouraged the synthesis of PGE_2_ and matrix degrading enzymes [26, 27].

Joint swelling is an important sign of synovial inflammation in RA [2]. In our study, the RA rat models were effectively developed 14 days after the CFA injection, and then fed with GS, CS and GC. After 25 days of continuous intervention, we found that paw volume of rats in the GC group was significantly lower than the MT group, while the paw volumes of rats in the GS and CS groups fall in between the NT and MT groups and were indifferent with the NT group. This finding suggested that combined treatment with glucosamine and chondroitin sulfate may have the better effect on alleviating joint swelling in RA. In addition, TNF-α, IL-1β, IL-10, PGE_2_, MMP-3 and NO were determined on day 50 of the CFA injection (i.e., D_36_). We discovered that levels of six serum inflammatory factors in the MT group were not different from the NT group, and also could not be influenced by GS, CS, or GC interventions. However, data from previous investigation indicated that levels of these serum inflammatory factors were increased on day 28 of the CFA injection [28, 29]. Inflammation in CFA-induced RA model rats would recede gradually [30]. The inconsistence of levels of inflammatory factors after CFA injection between our findings and others’ may be caused by the different sampling time point. In synovial tissue, however, pathology scores and expression levels of TLR-4 and NF-κB in the MT group were discovered to be higher than in the NT group and were reduced after GC intervention, suggesting that oral supplementation with GC could alleviate local inflammation levels in the joints in RA rats. We also found no significant differences in pathology scores and expression levels of TLR-4 and NF-κB in either the GS or CS groups compared with the MT group. Notably, pathological scores and expression levels of TLR-4 and NF-κB in both the GS and CS groups were not different from those in the NT group. Our findings indicated that GS and CS interventions also could alleviate the local inflammation in RA rat joints to some extent.

Furthermore, interactions between the intestinal microbiota and the host immune system were important in regulating the inflammatory state, and the disordered gut microbiota has been identified to trigger the inflammation [31, 32]. An epidemiological data have shown that clinical or subclinical symptoms of gut microbiota dysbiosis are prevalent in some RA patient [33]. Studies have shown that both GS and CS could influence the intestinal microbial composition, including RA-associated bacteria such as *Prevotella*, Bacteroidaceae, and Clostridiaceae [8–11]. We discovered that the intestinal microbiota composition in rats in the MT group differed from that in the NT group at D_18_ and D_25_ using PCoA and Anoism analysis, indicating that the AA model rats had intestinal microbiota dysbiosis. Compared with the MT group, no difference was seen in the intestinal microbiota composition in rats in either the GS or CS groups, but the GC group showed significant differences, which suggested that GC could improve bacteria dysbiosis. Intriguingly, we also found that GS, CS, and GC improved gut microbiota dysbiosis at D_18_, while joint swelling did not improve until D_25_, implying that gut microbiota dysbiosis generated by CFA injection recovered faster than joint swelling after GS, CS, and GC interventions.

LPS derived from bacteria had been demonstrated to cause arthritis and chronic inflammatory disorders via the TLR-4/NF-κB signaling pathway [12, 34]. We observed that LPS-producing bacteria such as *Enterobacter* [35] was more abundant in the MT group than in the NT group and was dramatically reduced after GC intervention at D_18_. Similarly, *Lactobacillus* [22, 23] and *Parabacteroides* [36], which were negatively related to LPS generation, were significantly decreased in the MT group compared to the NT group, and were significantly increased after GC intervention. Besides, we also discovered that GC group had lower abundance of some other LPS-producing bacteria, including *Erysipelotrichaceae_unclassified* [37], *Bacteroides* [38] and *Erysipelotrichaceae_uncultured* [37] compared with the MT group. These findings suggested that GC intervention could reduce the population of LPS-producing bacteria, lowering LPS production and thereby alleviating inflammation-induced joint damage in RA. Furthermore, the combined results of Figs. 5 and 6 show that GC intervention reduced the abundance of genera (*Candidatus Saccharimonas*, and *Eubacterium_ruminantium_group*) positively associated with joint swelling in the MT group, reduced the abundance of genera (*NK4A214_group*, *Candidatus_Saccharimonas*, and *RF39_norank*) positively associated with pathological parameters in the MT group, reduced the abundance of genera (*Nosocomiicoccus*) negatively associated with the anti-inflammatory factor IL-10 in the MT group, reduced the abundance of genera (*Eggerthellaceeae_unclassfied*, and *Clostidia_vadinBB60_group_norank*) positively associated with the pro-inflammatory factors IL-1β and TNF-α in the MT group, and reduced the abundance of genera (*Eubacterium_oxidoreducens_group*) positively associated with NO in the MT group. This finding suggested that the abundances of RA clinical injury-related genera and inflammation-related genera, were both reduced by GC intervention.

Collectively, GC intervention may reduce RA-induced joint inflammation and alleviate joint swelling and damage by inhibiting LPS-producing bacteria, lowering LPS synthesis, and thereby suppressing the activation of the TLR-4/NF-κB pathway in joint tissues. However, further high-quality evidence-based medical proof is required to conclusively prove the efficacy and duration of GS, CS, and GC in improving RA. More comprehensive studies are necessary to confirm whether the change in the gut microbiota is the cause or the consequence in the improvement of joint symptoms.

## Conclusions

This study found that both GS and CS could reduce the symptoms of RA-related joint inflammation and swelling to some extent, with the effect of GC being more apparent, providing a theoretical foundation for expanding the usage of GS and CS. Furthermore, we discovered that the bacteria enriched in the RA model were mostly strongly correlative with pro-inflammatory cytokines, right paw volume, and pathological score using correlation analysis. After GS, CS, and GC intervention, these bacteria enriched in the RA model recovered, with GC having the most apparent beneficial impact. Notably, the gut microbiota dysbiosis could be recovered before the improvement of joint symptoms after the intervention. Our findings also indicated that GC might inhibit LPS-producing bacteria and the activation of the TLR-4/NF-κB pathway, thus alleviating RA-induced joint inflammation and ameliorating the joint swelling and injury.

## Supplementary Information

**Additional file 1**. **Figure 1** Change curve of body weight from D-14 to D36

**Additional file 2**. **Figure 2** Photos of joint swelling in each group of rats

## Data Availability

The datasets used and/or analysed during the current study are available from the corresponding author on reasonable request. The relevant sequenced data have been deposited into a public database. Study No. SRP419979.

## Abbreviations

GS: Glucosamine
CS: Chondroitin sulphate
GC: Glucosamine plus chondroitin sulphate
RA: Rheumatoid arthritis
AA: Adjuvant arthritis
CFA: Complete Freund’s adjuvant
NF-κB: Nuclear factor kappa B
TLR: Toll-like receptor
TNF: Tumor necrosis factor
IL: Interleukin
PGE_2_: Prostaglandin E_2_
MMP: Matrix metalloproteinase
NO: Nitric oxide
ELISA: Enzyme immunosorbent assay
H&E: Hematoxylin-eosin
OTUs: Operational taxonomic units
PCoA: Principal coordinate analysis
LEfSe: The linear discriminant analysis effect size
SEM: Standard error of the mean
LPS: Lipopolysaccharide
