# Supplementary figures and images for: Combined treatment with glucosamine and chondroitin sulfate improves rheumatoid arthritis in rats by regulating the gut microbiota
Source: Nutr Metab (Lond). 2023 Apr 4;20:22. doi: 10.1186/s12986-023-00735-2 (PMC10071728; doi:10.1186/s12986-023-00735-2)

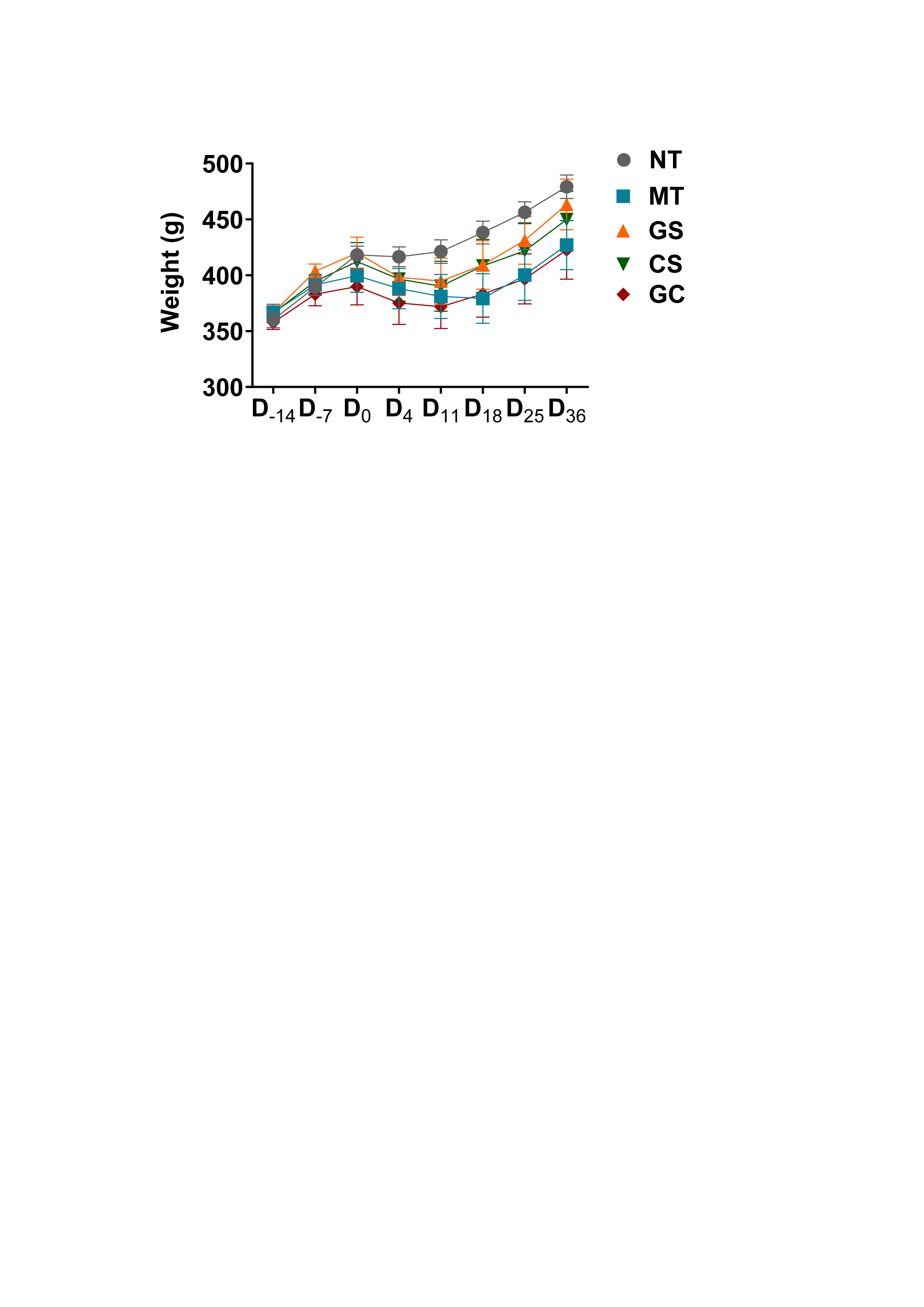

Supplement: Supplementary file 1 — Additional file 1. Figure 1 Change curve of body weight from D-14 to D36 [file 12986_2023_735_MOESM1_ESM.tif]

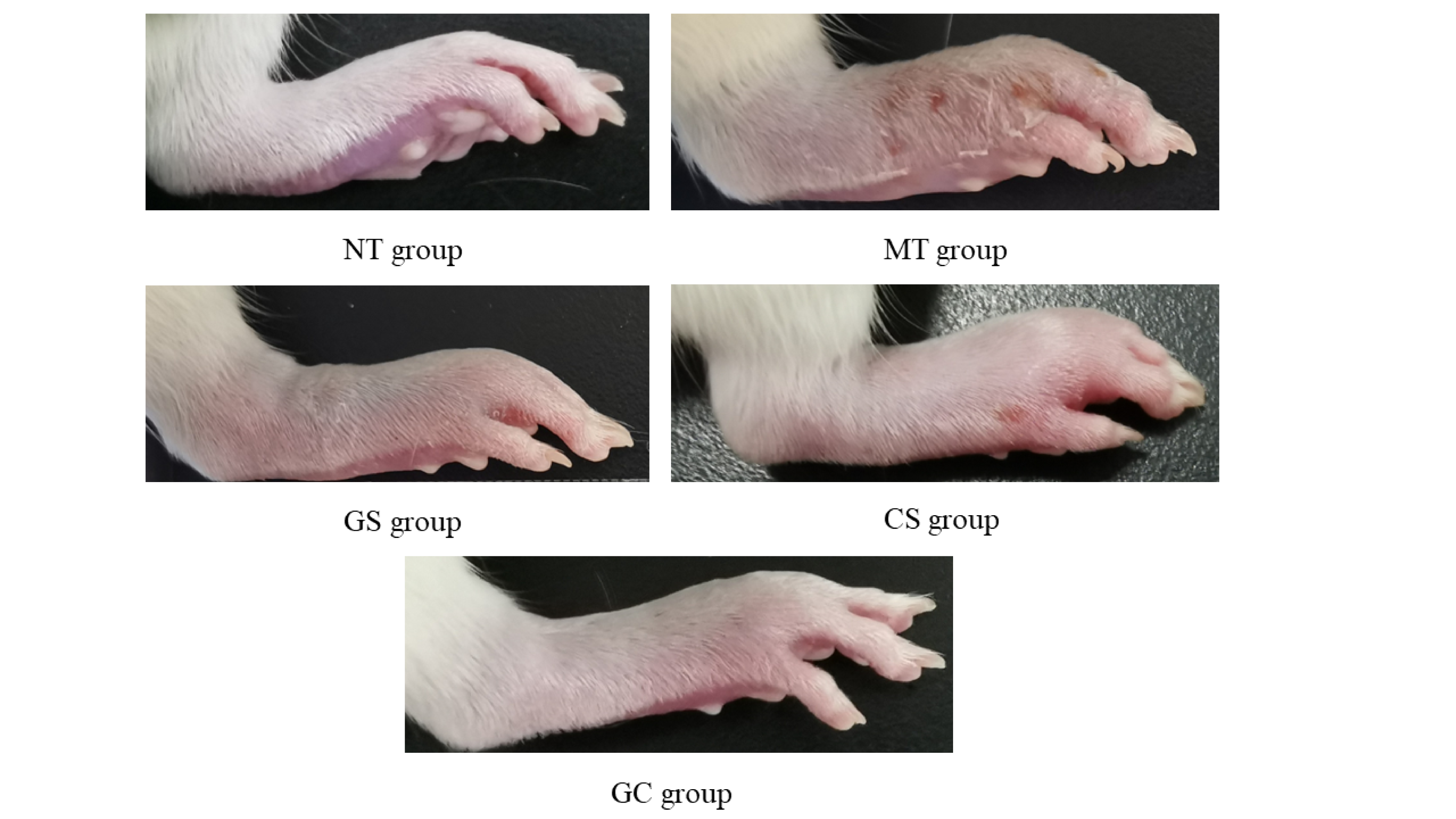

Supplement: Supplementary file 2 — Additional file 2. Figure 2 Photos of joint swelling in each group of rats [file 12986_2023_735_MOESM2_ESM.tif]
